# Socioeconomic Inequalities in Care Receipt at Older Ages: A Comparative European Study

**DOI:** 10.1093/geroni/igaa057.2566

**Published:** 2020-12-16

**Authors:** Ginevra Floridi, Ludovico Carrino, Karen Glaser

**Affiliations:** 1 King’s College London, London, United Kingdom; 2 King’s College London, London, England, United Kingdom

## Abstract

This study is among the first to investigate whether and how socioeconomic inequalities in the receipt of formal and/or informal care by disabled older adults vary across long-term care (LTC) systems. We link data from the SHARE survey with LTC system indicators for 136 regions in 12 European countries in 2015. Using multinomial multilevel models with cross-level interactions, we test whether and how income and wealth gradients in the receipt of only informal, only formal, and mixed care vary with the number of beds in residential LTC facilities across regions. We find pro-rich inequalities in the receipt of formal and mixed care only in regions with low or intermediate numbers of LTC beds, and no inequalities in regions with greater availability of residential beds. Our findings suggest that the presence of extensive formal LTC services may lead to a fairer distribution of formal and mixed care use across socioeconomic groups.

